# Electrochemical corrosion performance and microhardness of the electrodeposited Ni-CeO_2_ and Ni-ZrO_2_ nanocomposite coatings on the AA2219 aluminum alloy

**DOI:** 10.1039/d6ra00980h

**Published:** 2026-04-20

**Authors:** Sulaiman, Azim Khan, Naveed Zafar Ali, Shahzad Naseem, Saira Riaz, Sikander Azam, Ishaq Ahmad, Abdullah Jan

**Affiliations:** a Centre for Excellence in Solid State Physics, University of Punjab Lahore 54590 Pakistan shahzad.cssp@pu.edu.pk; b National Centre for Physics Islamabad 44000 Pakistan; c Zhejiang Provincial Key Laboratory for Cutting Tools, Taizhou University 318000 Zhejiang China azim@alum.imr.ac.cn; d International Joint Institute of Advanced Coating Technology, Taizhou University Taizhou 318000 P. R. China; e University of West Bohemia, New Technologies–Research Centre, 8 Univerzitní Pilsen 306 14 Czech Republic; f Abbottabad University of Science and Technology Abbottabad Pakistan; g Faculty of Engineering and Applied Sciences, Department of Physics, Riphah International University Islamabad Pakistan

## Abstract

Although Ni-based composite coatings have been widely studied, a systematic and direct comparison of the CeO_2_- and ZrO_2_-reinforced Ni nanocomposite coatings on AA2219 remains limited. Nickel-based nanocomposite coatings are widely used to enhance the surface hardness and corrosion resistance of the A2219 aluminium alloy in chloride-containing environments. In this work, to increase the durability of the AA2219 aluminium alloy, nickel nanocomposite coatings reinforced with varying contents of CeO_2_ and ZrO_2_ nanoparticles were fabricated on the alloy using composite electroplating. The results demonstrate that the uniform nanoparticle dispersion, particle chemistry, and optimized concentration (3.5 wt%) significantly enhance microhardness from 422 HV for pure Ni to 1089 HV for Ni-ZrO_2_ and 1055 HV for Ni-CeO_2_. Electrochemical corrosion resistance results for Ni-CeO_2_ show a significant reduction in corrosion current density (*I*_corr_) to 1.08 µA compared with 14.7 µA for Ni coatings, and charge-transfer resistance (*R*_cor_) of 25.1 kΩ compared with 0.386 kΩ for Ni coatings, corresponding to a significant enhancement and confirming the strong barrier effect of nanoparticle dispersion. Microstructural characterization and nanoparticle distribution were examined using transmission electron microscopy (TEM), scanning electron microscopy (SEM), and energy-dispersive X-ray spectroscopy (EDS), revealing pronounced grain refinement induced by both CeO_2_ and ZrO_2_ nanoparticles. The key scientific contribution of this study lies in the direct, mechanism-based comparison of CeO_2_- and ZrO_2_-reinforced Ni coatings deposited under identical conditions, providing new insights into their respective strengthening and anticorrosion mechanisms. These results advance the current understanding of Ni-based composite coatings and provide a useful framework for designing high-performance protective coatings for aluminium alloys for future aerospace coating applications.

## Abstract 2

The Ni-CeO_2_ and Ni-ZrO_2_ nanocomposite coatings on the AA2219 alloy exhibit enhanced hardness and corrosion resistance through uniform nanoparticle dispersion, grain refinement, and barrier/passive film mechanisms.

## Graphical abstract

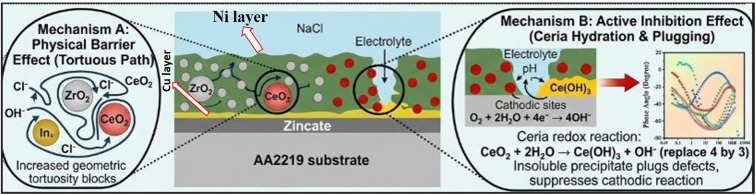

## 1 Introduction

Aluminium alloy AA2219 is a heat-treatable wrought alloy widely used in demanding engineering applications in the marine and aerospace industries due to its high strength-to-weight ratio, excellent weldability, and high fracture toughness. It exhibits reliable load-bearing performance over a wide temperature range.^1–3^ However, Al alloys are highly susceptible to galvanic and atmospheric corrosion because of the galvanic couple formation between CuAl_2_ intermetallic precipitates and the aluminium matrix. These precipitates tend to form during thermal processing and preferentially segregate along grain boundaries, which significantly accelerates corrosion, particularly in a chloride-rich environment.^4^ Although a high copper content (≈5.8–6.8 wt%) is essential for achieving superior mechanical strength and thermal stability, it simultaneously increases the corrosion susceptibility of the alloy.^5–7^ To address these challenges and extend the service life while enhancing mechanical performance and corrosion resistance, surface modifications have attracted considerable research attention.^2,8^ Material degradation is typically initiated at the surface. Electrodeposited metal–ceramic composite coatings are promising candidates for commercial applications in harsh and chemically aggressive environments. Nickel-based coatings, in particular, are widely employed due to their good corrosion resistance, strong adhesion to substrates, high hardness, and long-term reliability across various engineering fields.^9–11^ The Ni-electroplated coatings with the incorporation of ceramic or second-phase reinforcement particles, such as TiO_2_, SiC, CeO_2_, Al_2_O_3_, and ZrO_2_, have been shown to further enhance the mechanical properties and corrosion resistance. In particular, the dispersion of CeO_2_ nanoparticles in Ni matrices has been reported to significantly increase microhardness (up to ∼824 HV) compared with pure Ni, while also promoting grain refinement and dispersion strengthening, thereby improving corrosion protection through the formation of an effective passive barrier.^12^ M. Safavi *et al.*^10^ reported that the metal composite coatings exhibit significantly improved mechanical properties, wear resistance, and corrosion performance. The Ni-P coatings reinforced with CeO_2_ on a mild steel substrate show significant improvements in microhardness and corrosion resistance compared to those without the CeO_2_ dispersion Ni-P coatings.^13^ It has been reported that the corrosion and wear resistance of AA6060 alloy are significantly enhanced by the incorporation of ceria (CeO_2_) nanoparticles into electroplated Ni composite coatings. The resulting composite coating exhibited a microhardness of approximately 560 HV, representing a substantial improvement compared with ∼421 HV for pure Ni coatings. However, this strengthening effect is non-linear, as excessive nanoparticle content can lead to particle agglomeration, resulting in a rough and porous coating morphology that degrades mechanical integrity and overall coating performance.^14^ Most recently, Farough *et al.*^15^ successfully electrodeposited pure Co and Co/CeO_2_ composite coatings onto carbon steel substrates and systematically investigated the effects of CeO_2_ concentration, current density, and coating thickness on corrosion behaviour, mechanical properties, and wear resistance. Potentiodynamic polarization (PDP) results indicated that both pure Co and Co/CeO_2_ coatings exhibited less noble corrosion potentials compared with Ti–6Al–4V, while no pitting corrosion was observed. Electrochemical impedance spectroscopy (EIS) measurements further revealed a significant increase in total polarization resistance, reaching approximately 1540 Ω cm^2^ for pure Co and 1777 Ω cm^2^ for the Co/CeO_2_ composite coating, confirming enhanced corrosion protection. Moreover, the incorporation of CeO_2_ nanoparticles markedly improved the wear resistance of the composite coating compared with plain Co coatings and the uncoated carbon steel substrate. Zirconia nanoparticles with their well-dispersed characteristics enhance microhardness and corrosion resistance by promoting microstructural refinement and passive barrier effect, which is a more uniform and impermeable layer.^16^ A Ni-P coating with ZrO_2_ nanoparticle reinforcement on a copper substrate shows a significant increase in microhardness, which they correlated to the dispersion strengthening mechanism (Orowan strengthening) imparted by the hard ZrO_2_ particles, which explores the electrodeposition route for producing Ni-ZrO_2_ coatings.^17^ Pulse electrodeposited Ni-P-ZrO_2_ and Ni-W/ZrO_2_ nanocomposite coatings, in which ZrO_2_ nanoparticles have also been incorporated into PEO coatings on Ti–6Al–4V substrates, influence corrosion, antibacterial properties, and microstructure, also with decreased grain diameters, from 0.55 µm for pure Ni to 0.31 µm, and a significantly increased hardness, from 325 HV to 401 HV.^18^ Numerous studies have reported the incorporation of zirconia and ceria nanoparticles using various approaches to improve wear and corrosion resistance and mechanical properties. The existing literature lacks detailed studies to address the systematic and direct comparison of the dispersion of individual CeO_2_ and ZrO_2_ nanoparticles in Ni electroplating under the same deposition conditions, which produce galvanic cells that weaken the Ni coating. This work shows that the active redox nature of ceria provides excellent long-term protection and reduces galvanic drive more efficiently than the physical barrier provided by zirconia. In this study, the individual role of CeO_2_ and ZrO_2_ nanoparticles on grain refinement, improved hardness and corrosion of Al alloy are investigated. In addition, a significant improvement in microhardness was achieved due to controlled nanoparticle dispersion and the formation of a nanocrystalline Ni film, which therefore provides new insights into the comparative analysis of CeO_2_ and ZrO_2_ in Ni coatings, offering a simple and cost-effective approach to fabricate a predictive model to protect AA2219 alloy in chloride-rich environments Table 1.

**Table 1:** Electrodeposition parameters for the Ni-based nanocomposite coatings

Parameter	Value/description
Substrate (cathode)	AA2219 alloy
Anode	Pure Ni plate
Anode dimension	50 × 30 × 5 mm^3^
Inter-electrode gap	∼2 to 3 cm
pH	5.5–6.0
Current density	1.5 A dm^−2^
Bath temperature	40 °C
Deposition time	50–60 minutes
Magnetic stirrer	200 rpm

## 2 Experimental and characterization

### 2.1 Materials

Pure aluminium alloy AA2219 samples, with wt% compositions, as shown in Table 2, and dimensions of 15 × 10 × 2 mm^3^ were cut from the plate with purity >99%. A pure Ni plate was used as the anode material. The anode had a rectangular shape with dimensions of 50 × 30 × 5 mm^3^. ZrO_2_ and CeO_2_ nanoparticles were directly received from Macklin Company, Shanghai, China, as reported in our previous work. Analytical-grade chemicals, for the electroplating solution, including nickel sulfate hexahydrate (NiSO_4_·6H_2_O), boric acid (H_3_BO_3_), trisodium citrate dihydrate (C_6_H_5_Na_3_O_7_·2H_2_O), and sodium chloride (NaCl), were purchased from Macklin Biochemical Co., Ltd, Shanghai, China.

**Table 2:** Composition of the aluminum alloy (AA2219)

Aluminium (Al)	Copper (Cu)	Manganese (Mn)	Silicon (Si)	Titanium (Ti)	Vanadium(v)	Zinc (Zn)	Iron (Fe)
91.5 to 93.8 wt%	5.8 to 6.8 wt%	0.2 to 0.4 wt%	0.2 wt% max	0.02 to 0.10 wt%	0.05 to 0.15 wt%	0.03 wt% max	0.05 to 0.15 wt%

### 2.2 Fabrication of nanocomposite coatings on AA2219 alloy

**Fig. 1:**
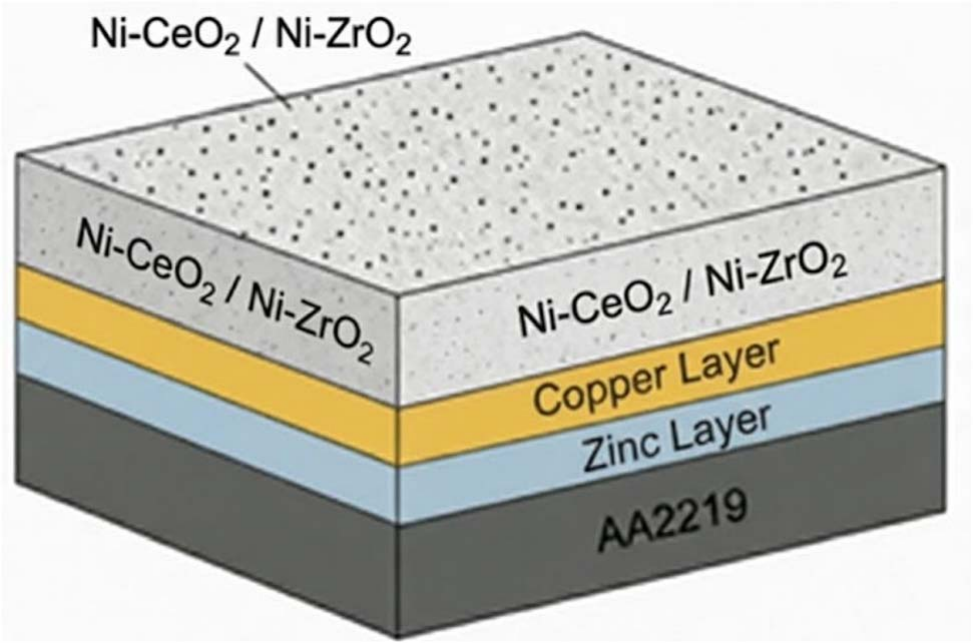
Schematic of the Ni-coated samples with CeO_2_ and ZrO_2_ nanoparticles on the AA2219 alloy.

Pure aluminum alloy AA2219 samples with dimensions of 15 × 10 × 2 mm^3^ were cut from the plate with purity >99%, polished with SiC paper, starting from 320 to 1200 grit, and cleaned ultrasonically with ethanol and acetone. The procedure for the surface treatment of an aluminium alloy prior to Ni electroplating involves several key steps to ensure a uniform and strongly adherent coating.^19,20^ The primary challenge is overcoming the natural surface oxide film on aluminium, which prevents proper plating. The sample is immersed in an alkaline solution. After alkaline cleaning, the specimen is rinsed and then immersed in a nitric acid solution (*e.g.*, 65% HNO_3_ for 5 seconds) to neutralize any remaining alkaline residues and to activate the surface. The double zincate process deposits a thin, uniform, and adherent layer of zinc onto the aluminium surface, which acts as a conductive layer and provides a compatible base for the subsequent nickel plating. The specimen is first immersed in a zincate bath solution for 45 s, rinsed and again immersed in the zincate bath solution for 15 s at room temperature, then dried by using a mechanical drier. After the zincate treatment, a thin copper layer is electroplated on the specimen in a copper bath at room temperature without magnetic stirring. The deposition time is 25 min with the same parameters as Ni electroplating, as shown in Table 1. The sample is again dried using a mechanical drier, and subsequently, the sample is ready for electrodeposition.^21^ Nanocomposite Ni films were electrodeposited on an AA2219 substrate at a current density of 1.5 A dm^−2^ using a bath containing 150 g l^−1^ NiSO_4_·6H_2_O, 35 g l^−1^ H_3_BO_3_, 120 g l^−1^ C_6_H_5_Na_3_O_7_·2H_2_O, and 12 g l^−1^ NaCl. ZrO_2_ and CeO_2_ nanoparticles were added separately to the bath at concentrations ranging from 5 to 10 g l^−1^. Then, samples with different wt% of Ni-ZrO_2_ or Ni-CeO_2_ nanocomposite are prepared by the same procedure.^22–24^ The weight percentages of dispersed oxide particles in the Ni films were determined by EDS using the INCA software package attached to the SEM instrument. Fig. 1 shows a schematic of composite coatings deposited on the AA2219 alloy. The bath was maintained at 40 °C with a pH of 5.5–6.0. These nanoparticles were ultrasonically dispersed for 30 min at 35 kHz before electrodeposition,^25^ and the solution was continuously stirred at 200 rpm using a magnetic stirrer to prevent nanoparticle agglomeration. The interelectrode gap was ∼2 to 3 cm.^26–30^ The electroplated samples were washed with acetone/ethanol, distilled water, and dried with a mechanical drier. The complete list of electrodeposition parameters for the Ni-based nanocomposite coatings is shown in Table 1.

### 2.3 Characterization

Field-emission scanning electron microscopy (FE-SEM, Inspect F50, Holliboro) was used to analyze the surface morphology of the as-prepared nanocomposite coatings. SEM/EDS results confirmed the presence of nanoparticles and quantified the weight percentage of dispersoids after electroplating. Transmission electron microscopy (TEM, JEM-2100F, JEOL, Japan) was used to confirm the size of the as-received nanoparticles. A Vickers microhardness tester (HMV Shimadzu company) was employed to measure microhardness of the layers. The measurements were performed at a load of 10 N for 10 s. The average of five measurements is presented. The electrochemical corrosion experiment was conducted using a Gamry electrochemical workstation, where the nickel-coated sample served as the working electrode, Ag/AgCl as the reference electrode, and a platinum mesh as the counter electrode. Tafel curves and impedance data of the coatings were obtained. The inter-electrode gap was maintained at approximately 5–10 mm, and the immersed surface area in the solution was about 1 cm^2^. The size of the platinum mesh electrode was 1 cm. Corrosion tests were conducted at room temperature using a working electrode with an area of 1 cm^2^ immersed in a 3.5 wt% sodium chloride (NaCl) solution at room temperature in a 60 mL solution with natural aeration. The sample was initially placed in the solution prior to the test to allow the open-circuit potential to stabilize before measurement. Tafel experiments were performed with a 4 mV s^−1^ scan rate, and electrochemical impedance spectroscopy (EIS) measurements were used to analyze the EIS at a frequency range of 0.1 Hz to 100 kHz. Tafel and EIS results of the Ni nanocomposite samples were analyzed by the Gamry Echem analyst software to determine the corrosion parameters. The coating thickness was experimentally confirmed in the laboratory using a LANDTEK digital meter, showing a thickness of approximately 20 µm. To confirm the accuracy of the results, the experiment was repeated three times.

## 3 Results and discussion

### 3.1 TEM results of the as-received nanoparticles and as-fabricated nanocomposite coatings

**Fig. 2:**
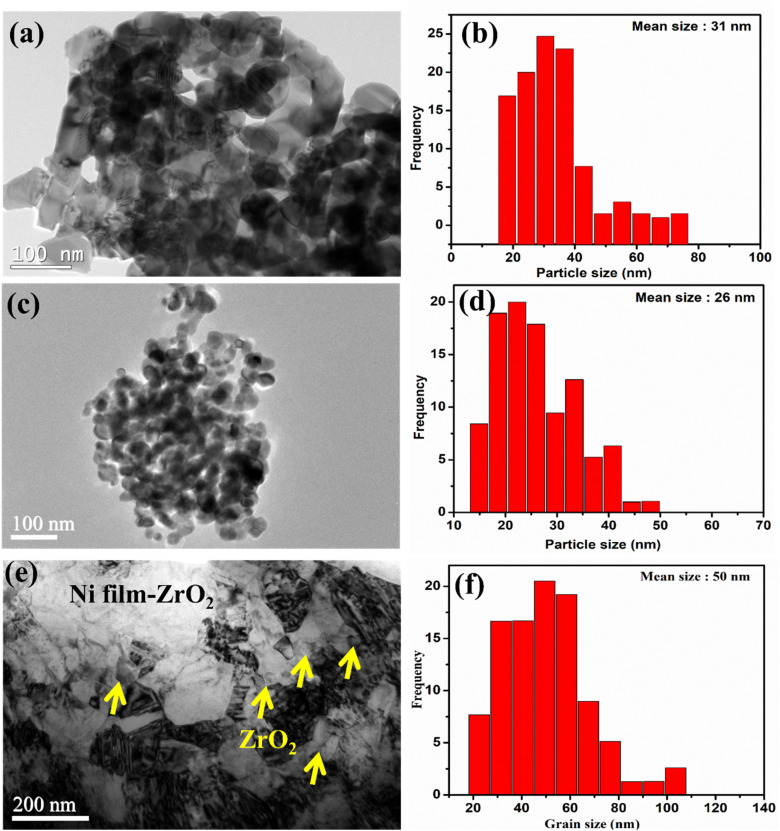
TEM images of the as-received CeO_2_ and ZrO_2_ nanoparticles (a and c), respectively, and their corresponding particle sizes (b and d). (e) TEM image of the Ni film with dispersed ZrO_2_ nanoparticles and (f) the corresponding grain size distributions.

Fig. 2 displays the as-received CeO_2_ and ZrO_2_ nanoparticles and their size distributions, along with the microstructure and grain size of the Ni nanocomposite film with dispersed ZrO_2_ nanoparticles. TEM results confirm the morphologies of the as-received CeO_2_ and ZrO_2_ nanoparticles, as shown in Fig. 2(a) and (c), respectively. The TEM analysis displays that ZrO_2_ nanoparticles exhibit smaller and more homogeneous particle sizes, whereas CeO_2_ nanoparticles appear as irregular and partially agglomerated clusters. Furthermore, the average particle sizes of CeO_2_ and ZrO_2_ nanoparticles were calculated to be approximately 31 nm and 26 nm, respectively, using the Nano measurer software, as shown in Fig. 2(b) and (d). Fig. 2(e) shows the surface microstructure of the as-prepared Ni nanocomposite film obtained from TEM analysis. The surface microstructure confirms that no cracks, cavities, or pores are present in the coatings. However, some twin structures were observed within the Ni film grains. The results also demonstrate a uniform distribution of ZrO_2_ nanoparticles throughout the nanocomposite Ni film, as indicated by the yellow arrows as shown in Fig. 2(e). In addition, localized agglomeration of ZrO_2_ nanoparticles can be observed in certain areas, as shown in Fig. 2(e). To further confirm the grain size of the nanocomposite Ni film with dispersed ZrO_2_ nanoparticles, the average grain size was measured to be approximately 50 nm using the Nano measurer software, as shown in Fig. 2(f). In a previous work by our group,^31^ it was reported that Ni films with dispersed CeO_2_ nanoparticles exhibited an average grain size of approximately 61 nm, whereas pure Ni films showed an average grain size of around 91 nm, which is significantly larger than that of Ni films with CeO_2_ and ZrO_2_ nanoparticle dispersions. More recently, the authors reported^29^ that pure Ni films exhibited an average grain size of approximately 100 nm. The dispersion of CeO_2_ and ZrO_2_ nanoparticles leads to pronounced grain refinement. This grain refinement is attributed to the freshly co-deposited nanoparticles, which suppress the growth of Ni grains along their preferred growth directions and/or promote the nucleation of new grains.

### 3.2 SEM/EDS surface morphologies of as-prepared Ni film with dispersed ZrO_2_ nanoparticles

**Fig. 3:**
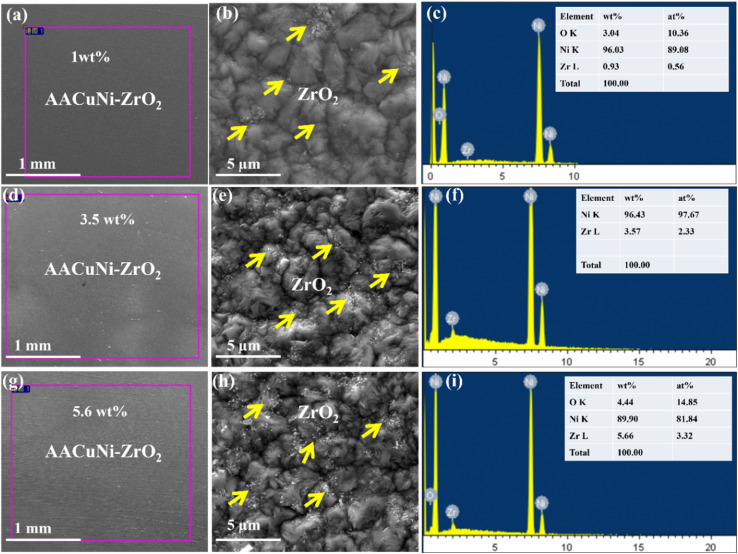
Surface morphologies and the corresponding EDS results of as-prepared Ni-ZrO_2_ nanocomposite coatings deposited on the AA2219 alloy: (a, d, and g) SEM images under low magnification and (b, e, and h) SEM images under high magnification of ZrO_2_ particle concentrations of 1 wt%, 3.5 wt%, and 5.6 wt%, respectively; and (c, f, and i) the corresponding EDS spectra.

Fig. 3 displays the surface SEM/EDS results of the as-prepared Ni nanocomposite films with various concentrations of dispersed ZrO_2_ nanoparticles. The surface microstructure of the Ni film with dispersed ZrO_2_ with a low content of approximately 1 wt% deposited on the Al alloy is shown in Fig. 3(a). The corresponding high-magnification image clearly reveals the dispersion of ZrO_2_ nanoparticles, as indicated by the yellow arrows in Fig. 3(b).

The EDS analysis taken from the surface region marked by the purple square in Fig. 3(a), which confirms the presence of approximately 1 wt% ZrO_2_ nanoparticles, is shown in the EDS spectrum in Fig. 3(c). The elemental composition was quantified using the INCA software attached to the SEM/EDS system. Multiple samples were prepared and examined by SEM/EDS, and appropriate samples were selected for further experiments. The particle concentration was increased to 3.5 wt%, and the resultant surface microstructure of the Ni nanocomposite film is shown in Fig. 3(d). The high-magnification image (Fig. 3(e)) confirms the uniform dispersion of ZrO_2_ nanoparticles, as indicated by yellow arrows. The increased nanoparticle content is clearly reflected in the surface microstructure. The corresponding SEM/EDS spectrum further confirms the wt% of ZrO_2_ nanoparticles in the nanocomposite film, as shown in Fig. 3(f). The ZrO_2_ nanoparticle content was further increased from 3.5 wt% to 5.6 wt%, and the resultant surface morphologies and EDS results are presented in Fig. 3(g–i). The surface microstructure clearly indicates a higher concentration of nanoparticles, as highlighted by yellow arrows in Fig. 3(h). At this higher wt%, a small degree of particle agglomeration can also be observed. The EDS spectrum confirms the elemental wt% in the nanocomposite film, as shown in Fig. 3(i). The results indicate the successful and uniform dispersion of ZrO_2_ nanoparticles at various concentrations (1 wt%, 3.5 wt%, and 5.6 wt%) in the nanocrystalline Ni film, without cavities, pores, or cracks.

### 3.3 Microstructures and the corresponding EDS results of the as-prepared Ni, Cu–Ni, and CeO_2_-dispersed coatings on Al alloy

**Fig. 4:**
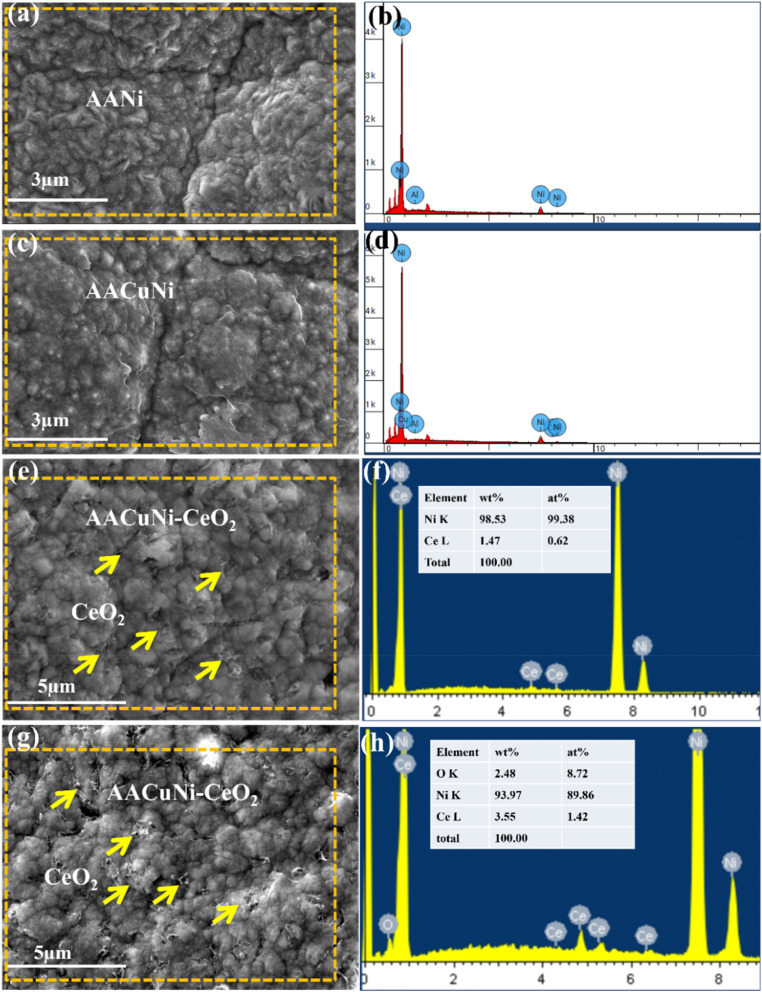
Surface microstructures and the corresponding EDS results of the as-prepared coatings on the Al alloy. Surface morphologies of (a) AA Ni coating, (c) AACuNi, and (e and g) AACuNi-CeO_2_ nanoparticle-dispersed coatings. (b), (d), (f) and (h) present the corresponding EDS spectra, confirming the elemental composition of the coatings.

Fig. 4 illustrates the surface microstructures and the corresponding EDS results of coatings prepared on the Al alloy using composite electroplating. The AANi and AANiCu samples shown in Fig. 4(a–c) present clear surface morphologies in the magnified images. The corresponding EDS results are shown in Fig. 4(b–d). Fig. 4(e–g) show the surface morphologies of AACuNi coatings with dispersed CeO_2_ nanoparticles at concentrations of 1.4 wt% and 3.5 wt%, respectively. The corresponding SEM/EDS analyses confirm the nanoparticle concentrations, as shown in Fig. 4(f–h). The surface microstructures clearly evidence the presence of CeO_2_ nanoparticles, as indicated by yellow arrows in Fig. 4. It is also evident from the surface morphologies that the coatings with the CeO_2_ nanoparticle dispersion exhibit a more compact structure with finer grain morphologies compared to the coatings without the nanoparticle dispersion. This observation is consistent with the TEM results shown in Fig. 2(e), where a smaller grain size of approximately 50 nm is observed. In our previous work, the grain size of CeO_2_-dispersed coatings was reported to be around 61 nm, compared to approximately 100 nm for the pure Ni film reported in our recent study.^29,32^

Generally, it is anticipated that combining a dense nickel matrix with particle reinforcement (ZrO_2_ or CeO_2_) will increase corrosion resistance primarily through enhancing route flexibility for corrosive species, introducing chemically stable inclusions that prevent ion transport and fine grains promoting uniform passive-film formation. The presence and uniform dispersion of Ce, Zr, and O elements inside the Ni matrix are confirmed by the SEM/EDS spectra of Ni-CeO_2_ and Ni-ZrO_2_ coatings, as shown in Fig. 3 and 4. In addition to improving the coating compactness, the addition of CeO_2_ and ZrO_2_ offers unique corrosion protective mechanisms, as CeO_2_ provides active corrosion inhibition through Ce^3+^/Ce^4+^ redox transitions and as an oxide film promoter. Meanwhile, ZrO_2_ acts as a stable, inert barrier phase that prevents corrosive species from diffusing. Therefore, when compared to pure Ni coatings, the results demonstrate that both Ni-CeO_2_ and Ni-ZrO_2_ composite coatings exhibit better structural integrity and a higher potential for corrosion resistance. The nanoparticle dispersion improves enhanced grain refinement, which increases the surface to volume ratio. The resulting top passive layer of NiO and Ni (OH)_2_ (ref. 33 and 34) forms a smooth surface suitable for various industrial applications. The crack-free coatings prevent the penetration of corrosive ions in capillaries/pores/cracks, which enhances the anticorrosive protection performance of materials.

### 3.4 Micro-hardness

**Fig. 5:**
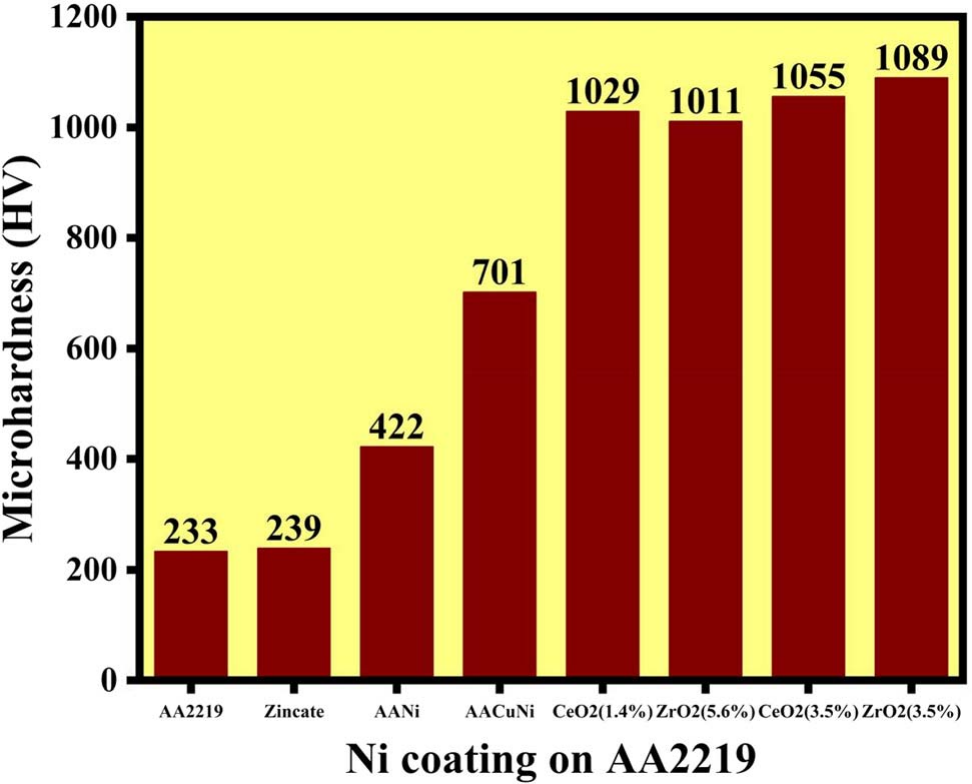
Microhardness plot of different wt% of CeO_2_ and ZrO_2_ nanoparticles in Ni coatings on the AA2219 aluminum alloy.

Surface modification by nickel electrodeposition provides significant enhancement in mechanical properties, particularly microhardness, for demanding structural applications to improve the performance, service life, and corrosion resistance of components. These nanocomposite coatings offer superior corrosion resistance and excellent mechanical performance through the incorporation of hard ceramic nanoparticles.^35^ In this study, we focus on the effects of incorporating different wt% zirconia (ZrO_2_) and ceria (CeO_2_) nanoparticles in nickel electroplating on AA2219 substrates. Microhardness of the Ni nanocomposite coating depends on various factors like strengthening the interface between Ni nanoparticles, grain refinement, nanoparticle distribution, reinforcement effect, and substrate/coating adhesion. These hard, non-deformable nanoparticles are uniformly distributed within the FCC Ni matrix, forming a compact coating (see Fig. 2(e), 3, and 4). Their presence affect the microstructure of the Ni coatings, which restrict grain growth and lead to grain refinement, as confirmed by TEM (Fig. 2(e)). This refinement significantly enhances hardness *via* the Hall–Petch effect. Both these nanoparticles act as nucleation sites during coating and provide a more compact and nanoscale structure,^36,37^ thus blocking the dislocation motion and plastic deformation in the nickel matrix, which is explained by the Orowan strengthening process and the primary agents of plastic deformation in crystalline materials have to bend around unshearable nanoparticles.^38^ A uniform dispersion is achieved by controlled magnetic stirring and ultrasonic agitation. Grain refinement enhances the hardness of nanocomposite coatings. High current density decreases coating adhesion; therefore, the adhesion is the highest at 1.5 A dm^−2^, as confirmed in the literature.^29,39^ During electrodeposition, a nanoparticle content of 3.5 wt% showed a significant improvement in mechanical properties, hardness and corrosion performance of coatings. Zirconia nanoparticles are inert, hard particles that promote a more compact coating structure. The resulting grain refinement, as shown in Fig. 2 based on the TEM results, leads to changes in surface morphology and increases surface density, which is one of the reasons for the higher hardness of the zirconia-containing coatings compared with ceria-containing coatings. In this study, the electrodeposition of ZrO_2_ led to a substantial increase in the hardness of the Ni coating, from 422 ± 8 HV to 1089 ± 9 HV, demonstrating a remarkable enhancement in the hardness of Ni electrodeposited on the aluminum alloy, as shown in Fig. 5. Similarly, the incorporation of CeO_2_ also resulted in a significant hardness improvement, reaching 1055 ± 11 HV, which is only slightly lower than that achieved with the dispersion of ZrO_2_ nanoparticles. This optimum nanoparticle content (∼3.5 wt%) shows a beneficial effect and prevents the nanoparticles from agglomeration, which leads to the degradation in mechanical properties at higher and much lower loadings, as shown in Fig. 5. When used individually, both ceramic nanoparticles produced pronounced hardness enhancement, along with improved corrosion resistance. In contrast, most reported studies show comparatively lower improvements in hardness, even with the addition of other hardening elements such as Co or W.^40^ The improved adhesion of successive coatings is achieved through the double zincating (DZ) process, which includes nitric acid etching and generates a micro/nanoporous Al surface with finer Zn nucleation, as reported previously (Table 3). The electroplated Cu interlayer promotes the formation of CuO/ZnO phases that suppress Al_2_O_3_ formation and act as a compact metallic barrier, thereby reducing charge transfer, ion transport, and oxygen permeability.^41^ Although the protective Cu oxide layer may partially dissolve under high chloride conditions, the Cu interlayer still enhances hardness, provides a crack-barrier effect, and improves long-term mechanical stability when the Ni layer is damaged. However, excessive nanoparticle agglomeration can degrade coating integrity and reduce the hardness of coatings.^42,43^

**Table 3:** Microhardness values of different wt% of the CeO_2_ and ZrO_2_ nanoparticles in Ni coating on the AA2219 aluminum alloy

Sample	AA2219	Zincate	AANi	AACuNi	CeO_2_ (1.4%)	ZrO_2_ (5.6%)	CeO_2_ (3.5%)	ZrO_2_ (3.5%)
Hardness (HV)	233 ± 4	239 ± 4	422 ± 8	701 ± 12	1029 ± 16	1011 ± 14	1055 ± 11	1089 ± 9

### 3.5 Electrochemical characterization

Electrochemistry plays important role in various fields, including batteries, water splitting, corrosion studies and fuel cells. Material durability and anticorrosion properties are determined by oxidation-reduction redox reactions on the metal surface.^44^ Electrochemical behavior of Ni nanocomposite coatings, without and with nanoparticles (ceria and zirconia) on AA2219, is investigated in a 3.5% NaCl solution to study the corrosion resistance under corrosive and harsh environment conditions.

#### 3.5.1 Potentiodynamic polarization

**Fig. 6:**
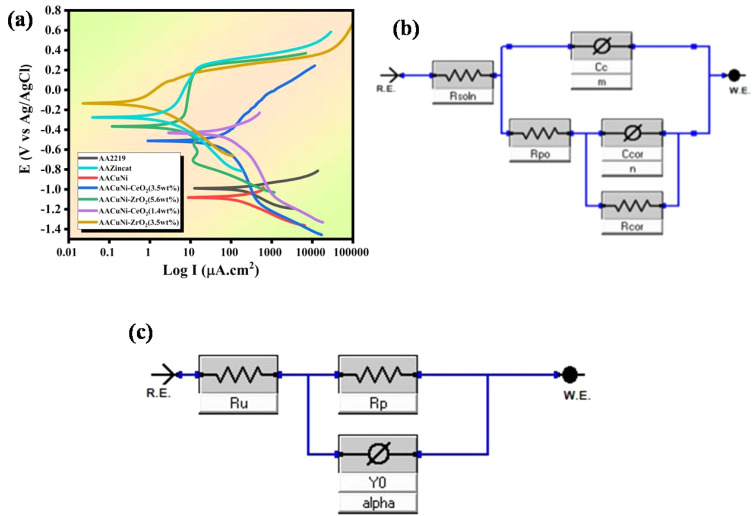
(a) Tafel curves of the Ni-coated samples with different wt% of the CeO_2_ and ZrO_2_ nanoparticles. (b) Equivalent reap2cpe circuit model. (c) Equivalent cpe circuit model.

**Fig. 7:**
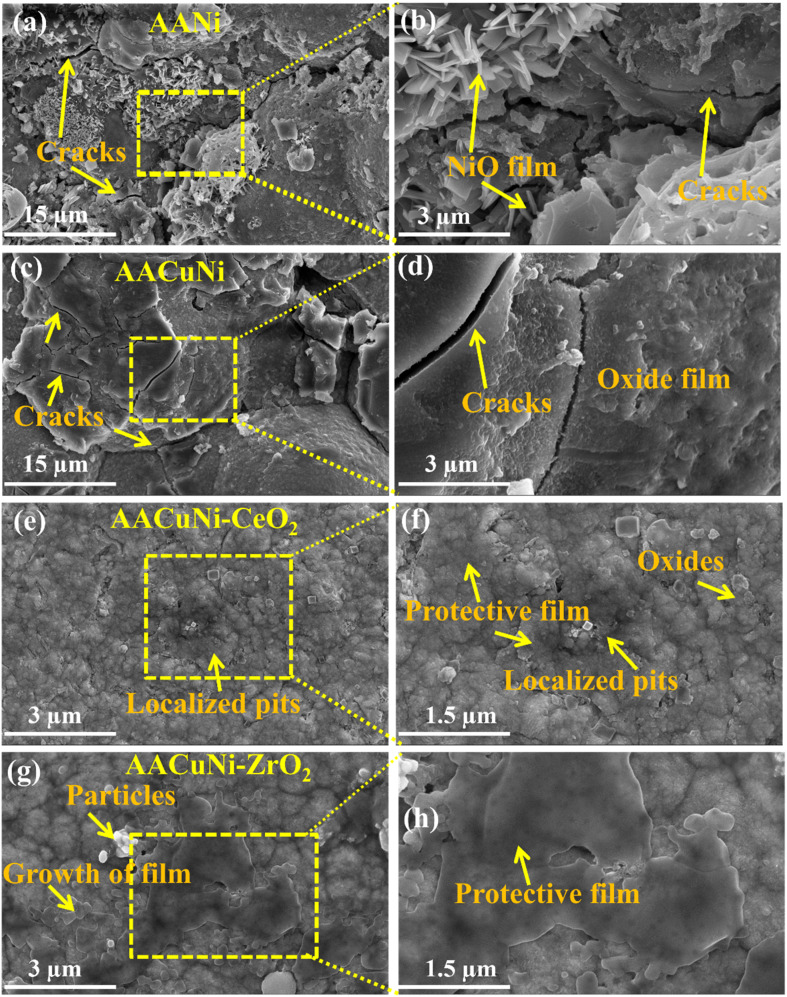
(a), (c), (e), and (g) SEM surface morphologies of the Ni film without and with dispersed CeO_2_ and ZrO_2_ nanoparticles on the AA2219 alloy, after corrosion in 3.5% NaCl solution. (b), (d), (f), and (h) Corresponding magnified images.

The Tafel extrapolation method was utilized to examine the electrochemical measurements, as shown in Table 4. AA2219 is inherently prone to corrosion, and the electrodeposition of nickel nanocomposites provides microstructural refinement and densification, establishing a robust physical barrier that significantly enhances corrosion resistance. The corrosion potential (*E*_corr_) represents the equilibrium potential at which the material neither corrodes nor passivates.^45^ The Ni-CeO_2_ coating exhibits a significant increase in corrosion potential (*E*_corr_ = −133 mV) compared to pure Ni (*E*_corr_ = −509 mV) and Ni-ZrO2 (*E*_corr_ = −368 mV) coatings, shifting to a more positive value, as shown in Fig. 6(a). The observed shift signifies a reduced thermodynamic susceptibility to corrosion, while the intermediate copper layer significantly enhances the overall corrosion resistance. All the coated samples demonstrate high corrosion resistance because of low corrosion current density (*I*_corr_), which reflects the rate of the electrochemical reaction due to the addition of ceria nanoparticles, reducing *I*_corr_ from 14.70 µA to 1.80 µA. The *R*_p_ is inversely proportional to *I*_corr_, and a higher *R*_p_ indicates better anticorrosion properties.^46^ The Ni-CeO_2_ sample demonstrated enhanced protective performance, characterized by the lowest *I*_corr_ and highest polarization resistance. As shown in Fig. 6(a), Ni-CeO_2_ and Ni-ZrO_2_ exhibit superior resistance to localized corrosion, characterized by high pitting potentials and stable *I*_corr_.^47^ A passive film, as shown in Fig. 7(e–h), enhances the stability of the passive film and provides a robust barrier against chloride corrosive species, thereby minimizing the susceptibility to pitting corrosion; the homogeneous distribution of nanoparticles mitigates microstructural defects and obstructs aggressive ion transport, facilitating stable passivation and superior corrosion resistance.^48^ The optimum concentration of 3.5 wt% for both nanoparticles prevents agglomeration, resulting in an efficient crystalline structure and superior corrosion resistance, as verified by hardness and EIS studies. While the Ni-CeO_2_ and Ni-ZrO_2_ coatings with 1.4 wt% and 5.6 wt% nanoparticles, respectively, show lower corrosion current density, but lower than that of 3.5 wt% due to the agglomeration of high concentration nanoparticles, indicating good anti-corrosion characteristics in a 3.5 wt% NaCl solution at 25 °C.

**Table 4:** Tafel parameters obtained by the fitting of the Tafel curves of the Ni nanocomposite samples

Sample	Beta A mV per decade	Beta C mV per decade	*I*_corr_ (µA)	*E*_corr_ (mV)	Corrosion rate (mpy)	Resistance polarization (KΩ)	Corrosion efficiency (*η*_p_%)	Chi squared (*χ*^2^)
AA bare	74.8	209	200.0	−991.0	57.46	0.12		0.12
Double zincate	94.9	253.7	165.0	−1080	14.21	0.18	17.5	0.1
AACuNi	166.5	209.1	14.70	−509.0	7.2	2.73	92.65	0.05
AACuNi-ZrO_2_(3.5 wt%)	222.0	639.6	5.600	−368.0	2.228	13.05	97.2	0.01
AACuNi-ZrO_2_(5.6 wt%)	80.0	87.7	26.50	−432.0	10.56	1.29	86.75	0.026
AACuNi-CeO_2_(1.4 wt%)	555	250.6	3.27	−275	1.303	1.88	98.3	0.049
AACuNi-CeO_2_(3.5 wt%)	471.2	272.1	1.08	−133.0	0.431	69.35	99.4	0.08

#### 3.5.2 Surface microstructure analysis after corrosion in 3.5 wt% NaCl solution

Fig. 7 shows the surface morphology of Ni films without and with nanoparticle dispersion after corrosion on an Al alloy in a 3.5 wt% NaCl environment. The AANi coatings exhibit severe corrosion, characterized by corrosion pits, microcracks (indicated by yellow arrows), and a non-uniform corrosion product layer, as shown in Fig. 7(a). Furthermore, it is confirmed that the formed oxide or hydroxide film is loosely adhered to the coating surface. The magnified image more clearly shows the significant growth of corrosion products and microcracks, along with the formation of an oxide film (NiO) layer, as presented in Fig. 7(b). This behavior is attributed to galvanic coupling between Ni and Al and the easy penetration of chloride ions through microcracks and pores, leading to unstable Ni passive films for Ni coatings on Al substrates in NaCl environments. The introduction of a Cu interlayer significantly improves corrosion resistance^49^ by reducing the galvanic mismatch between Al and Ni and enhancing the formation of a more adherent corrosion film, as shown in Fig. 7(c), in comparison with the coating without a Cu layer (Fig. 7(a)). However, cracks and corrosion product layers are still observed in the chloride environment. In such conditions, Cu oxides/hydroxides may partially dissolve, allowing localized corrosion to initiate at coating defects in the 3.5 wt% NaCl solution.

The Ni film with dispersed CeO_2_ nanoparticles shows further improvement. CeO_2_ nanoparticles provides active corrosion inhibition through the Ce^3+^/Ce^4+^ redox couple, promoting re-passivation and suppressing cathodic oxygen reduction.^50,51^ The surface passivation layer becomes smoother and more adherent, with only a few localized pits, as indicated by the yellow arrow in Fig. 7(e). These minor localized pits may be associated with nanoparticle agglomeration, which can be observed more clearly in the magnified image shown in Fig. 7(f). The AACuNi-ZrO_2_ coatings exhibit the best corrosion resistance, characterized by a more compact and adherent oxide layer (Fig. 7(g)) and suppressed crack formation. ZrO_2_ is chemically inert and does not participate in redox reactions; instead, it provides a strong physical barrier protection. The dispersion of ZrO_2_ nanoparticles causes significant grain refinement of the Ni matrix, which enhances passivity, reduces defect size, and promotes the formation of a uniform and stable NiO/Ni(OH)_2_ film. Fine grains also shorten diffusion paths for re-passivation, effectively preventing chloride penetration into the substrate and contributing to the formation of a thicker and more protective surface layer, as clearly observed in the magnified image (Fig. 7(h)) and presented in Fig. 9, as a schematic of the corrosion mechanism.

#### 3.5.3 Electrochemical impedance spectroscopy (EIS)

Electrochemical impedance spectroscopy (EIS) is used to evaluate corrosion resistance by analyzing the coating–electrolyte interface. The key parameters include *R*_s_ (electrolyte resistance), *R*_ct_ (charge transfer resistance), *R*_c_ (coating resistance), *C*_dl_ (double layer capacitance), and *C*_c_ (coating capacitance), which reflects the dielectric properties of the coating layer. Higher *R*_ct_ suggests slow corrosion, while lower *C*_c_ indicates a coating with low water absorption, dielectric constant, and high barrier characteristics. For marine and aerospace corrosion resistance, immersion tests in 3.5 wt% NaCl are utilized. Impedance modulus indicates corrosion resistance; larger semicircles or longer arcs signify high corrosion resistance.

**Fig. 8:**
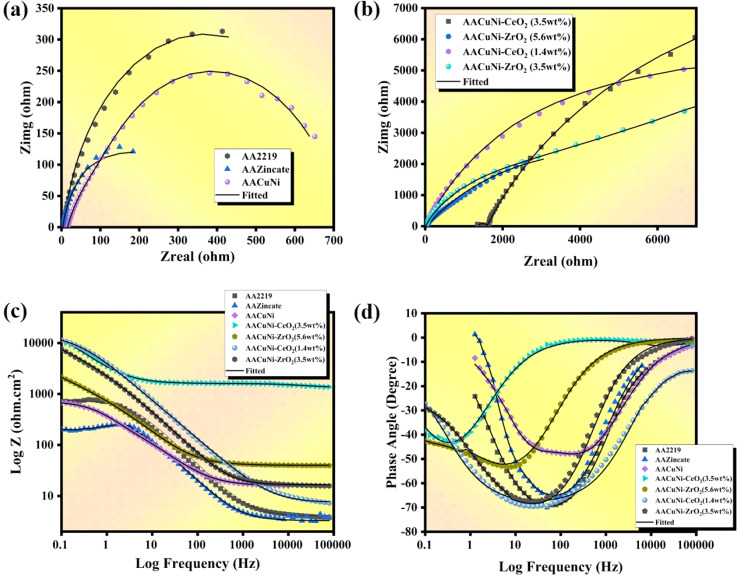
(a) Nyquist plot of the Ni film without nanoparticles. (b) Nyquist plot of Ni-CeO_2_ and Ni-ZrO_2_ with different wt%. (c) Impedance plot of the Ni nanocomposite. (d) Phase angle plot of the Ni Nanocomposite.

An equivalent circuit fitting model (reap2cpe) for Ni films with and without the nanoparticles, as shown in Fig. 6(b), and an equivalent circuit fitting model (cpe) for the bare sample, as shown in Fig. 6(c), were used to analyze the EIS data. Also, the data is well-fitted, as shown in Fig. 8. As demonstrated in Fig. 6(a), adding CeO_2_ to the protective coating significantly increases the corrosion resistance. The formation of a passive stable layer on the Ni coating, where ceria works similarly to what is stated in the Tafel plot, thereby limiting anodic dissolution and Cl^−^ ion entry, is one of the microstructural and morphological factors responsible for the improvement in stability.^45,50,52^ FESEM images verify that ceria and zirconia nanoparticles seal surface pores, forming a homogeneous, dense structure that prevents corrosive agents and enhances the anticorrosive properties of the coating. Table 5 shows that Ni coating has a film resistance (*R*_po_) of 0.3 KΩ. With the increase of both ceria and zirconia contents, *R*_po_ increases from 0.301 KΩ to 5.03 KΩ, demonstrating that ceria and zirconia nanoparticles create a compact, uniform layer. This dense coating prevents water, oxygen, and corrosive ions from entering the metal substrate. The *R*_po_ values are confirmed by comparable results for *R*_cor_, which also increased from 0.3 KΩ to 25.1 KΩ, while the *C*_dl_ and *C*_c_ values decreased. Ni-CeO_2_ and Ni-ZrO_2_ with the optimal concentration of 3.5 wt% improve the coating's anticorrosive capabilities. Ceria nanoparticles provide a strong nickel oxide layer that prevents Cl^−^ ions.^53^ The *n* and *m* values are close to 0.9, showing a highly uniform coating, which is verified by the FESEM images. The Bode plot shows the phase angle (*ϕ*) and impedance magnitude (*Z*) in relation to the frequency of the AC signal (*f*), as shown in Fig. 8(c) and (d).

**Table 5:** EIS parameters obtained by the fitting of EIS curves of the Ni nanocomposite samples

Sample	*R*_soln_ (kΩ)	*R*_cor_ (kΩ)	*R*_po_ (kΩ)	*C*_cor_ (S s^*n*^)	*n*	*C*_c_ (S s^*n*^)	*m*	Thickness (mm)	Goodness of fit (*χ*^2^)
AA bare	4.575		0.555	175.5 × 10^−6^	0.95			2 ± 0.2	0.008
AA zincate	3.353	0.164	0.301	4.001 × 10^−6^	0.8	206.5 × 10^−6^	0.856	28 ± 0.4	0.004
AACuNi	15.76	0.386	0.328	263.2 × 10^−6^	0.9	381.3 × 10^−6^	0.755	53 ± 0.5	0.00025
AACuNi-ZrO_2_ (3.5 wt%)	16.20	19.1	2.72	105.2 × 10^−6^	0.5	70.34 × 10^−6^	0.833	22 ± 0.24	0.012
AACuNi-CeO_2_ (1.4 wt%)	7.45	20.8	4.1	42.5 × 10^−6^	0.8	52.16 × 10^−6^	0.775	21 ± 0.12	0.004
AACuNi-ZrO_2_ (5.6 wt%)	19.1	10.13	1.1	565.5 × 10^−6^	0.52	237.1 × 10^−6^	0.768	26 ± 0.2	0.001
AACuNi-CeO_2_ (3.5 wt%)	15.2	25.1	5.03	38.47 × 10^−6^	0.88	84.73 × 10^−6^	0.723	24 ± 0.1	0.00056

A strong correlation exists between corrosion resistance and the height of the curve (*Z*) across the frequency range. This plateau value is proportional to polarization resistance (*R*_p_), which is inversely related to the corrosion rate. At mid-frequency, the line slope is related to the capacitive behaviour of the protective layer.^54^ Higher impedance, especially at low frequencies, indicated improved corrosion resistance. The zincate curve, which is the least resistant to corrosion, has the lowest impedance at low frequencies, whereas the Ni-CeO_2_ curve has the highest impedance compared to Ni-ZrO_2_. As shown in Fig. 8(d), the Ni nanocomposite curves exhibit a more complex pattern, suggesting two overlapping time constants, and the downward shifts in phase angle indicate coating degradation due to the penetration of corrosive species.^55^ Ni-CeO_2_ with 3.5 wt% nanoparticles exhibits a lower and broader phase angle peak around −50° at low frequencies. A large peak at a low frequency with a minimal phase shift typically signifies a highly stable barrier layer. The peak shifts in Ni-CeO_2_ to lower frequencies suggest an increased corrosion resistance. This EIS result confirms that both CeO_2_ and ZrO_2_ (3.5 wt%), along with the Ni coating, exhibit superior anticorrosive performance on AA2219, resembling the Tafel results.

Table 6 systematically compares the electrochemical performance parameters, including charge-transfer resistance (*R*_ct_/*R*_cor_), coating resistance (*R*_po_), corrosion current density (*I*_corr_), and test environment, of Ni-CeO_2_ and Ni-ZrO_2_ nanocomposite coatings in the present work with the previously reported nanocomposite coatings from the literature.^56,57^ The comparison clearly demonstrates that AACuNi-CeO_2_ and AACuNi-ZrO_2_ (3.5 wt%) coatings developed in this work exhibit high charge-transfer resistance and coating resistance among the Ni-based nanocomposite coatings evaluated in a 3.5 wt% NaCl solution, as discussed in detail above and in the discussion section. The high charge-transfer and coating resistance values indicate the formation of a highly effective barrier layer, resulting in superior corrosion protection.

**Table 6:** Comparative electrochemical performance of Ni-based nanocomposite coatings

Coating system	Substrate	Electrolyte	*R*_ct_/*R*_cor_ (kΩ)	*R*_po_/film resistance (kΩ)	*I*_corr_ (µA)	Year
Ni-ceramic nanocomposite	Al alloy	3.5 wt% NaCl	8–15	N/R	3–10	2023
Polymer/ceramic composite	Steel	NaCl	5–12	1–3	5–20	2023
Ni-based nanocomposite	Al alloy	3.5 wt% NaCl	10–18	2–4	2–6	2023
Ni-CeO_2_ composite	Steel	NaCl	12–20	3–4	2–5	2024
Oxide-reinforced metal coating	Al alloy	NaCl	6–14	1–3	4–12	2024
AACuNi-ZrO_2_ (3.5 wt%)	AA2219	3.5 wt% NaCl	19.1	2.72	5.60	This work
AACuNi-CeO_2_ (3.5 wt%)	AA2219	3.5 wt% NaCl	25.1	5.03	1.08	This work

## 4 Discussion

### 4.1 Effect of uniform nanoparticle dispersion and optimized wt% on the corrosion resistance of nanocomposite films

**Fig. 9:**
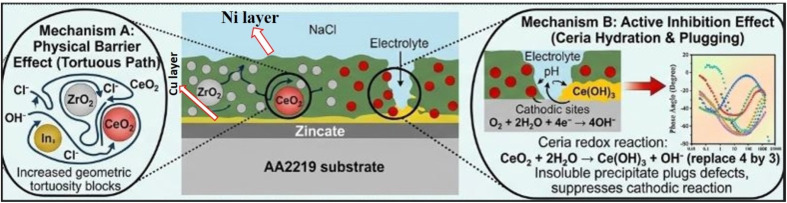
Schematic of the corrosion mechanism of the Ni coatings with dispersed ZrO_2_ and CeO_2_ nanoparticles on the AA2219 alloy.

The present work clearly shows that the optimal concentration with uniform dispersion of CeO_2_ and ZrO_2_ nanoparticles (around 3.5 wt%) significantly enhances the hardness and corrosion resistance of the Ni coating on AA2219 alloy. The uniform dispersion of nanoparticles is important because homogeneous distribution of CeO_2_ and ZrO_2_ nanoparticles hinders the movement of dislocations through the Orowan mechanism.^58^ The SEM/EDS results confirmed the uniform distribution of both nanoparticles within the Ni matrix without causing agglomeration, porosity, cracks, or cavities. These findings were further confirmed by TEM investigations. Furthermore, uniform dispersion results in a more compact and dense coating, which effectively inhibits the pathways for electrolyte (Cl^−^) penetration.^59^ This observation is also supported by electrochemical spectroscopy. The Tafel plot indicates a significant decrease in the corrosion current density (*I*_corr_), and a positive change in the corrosion potential (*E*_corr_) of both Ni-CeO_2_ and Ni-ZrO_2_ wt% coatings compared to the Ni coating without the dispersion of nanoparticles. Specifically, the Ni-CeO_2_ coating with 3.5 wt% content exhibits the lowest *I*_corr_ and the highest polarization resistance, yielding the most effective corrosion resistance. The EIS results, in which a drastic rise in the resistance of charge transfer (*R*_cor_) and coating resistance (*R*_po_) accompanied by a fall in coating capacitance, prove the barrier layer formation as being quite an effective anticorrosive layer. CeO_2_ nanoparticles prevent corrosion through a reversible Ce^3+^/Ce^2+^ redox pair.^50^ As a result of the dissolution of Ce^3+^ ions, CeO_2_ can be partially reduced to Ce_2_O_3_. When these cerium species migrate to the substrate surface, they are re-oxidized to Ce^4+^ due to their strong affinity for oxygen. This process promotes the formation of a stable cerium oxide layer on the surface, which acts as a protective barrier and enhances the anticorrosion performance of the coating.^60^ The schematic of this mechanism is shown in Fig. 9. The reversible Ce^3+^/Ce^4+^ redox couple enables rapid re-passivation and self-healing behavior.^50^ Due to this redox activity, ceria offers more effective corrosion inhibition than zirconia. ZrO_2_ nanoparticles provide a high anti-corrosivity because of blocking the pathways of diffusion of chloride ions, as well as retaining the passive NiO/Ni(OH)_2_ surface, and make it a chemically inert and physically strong barrier phase. The post-corrosion SEM images provide clear evidence that both nanoparticle-reinforced coatings exhibit significantly enhanced surface protection and fewer corrosion pits and microcracks compared to the pure Ni coating.

### 4.2 Grain refinement enhanced corrosion resistance and hardness

The CeO_2_ and ZrO_2_ nanoparticle dispersions act as heterogeneous nucleation sites in electrodeposition, thereby leading to increased coating density, as well as significantly enhanced grain refinement. This grain refinement reduces internal defects and microcracks that normally improve the corrosion resistance. By successfully blocking the diffusion pathways of aggressive species like Cl^−^ ions, the nanoparticles increase the complexity of corrosion pathways and slow down electrochemical reactions at the coating electrolyte interface.^50^ The TEM analysis shows that upon adding CeO_2_ and ZrO_2_ nanoparticles, the grain size was significantly refined up to 50 nm, compared to the Ni coating without nanoparticles. This grain refinement is attributed to the pinning effect of the co-deposited nanoparticles, which facilitates the nucleation of new grains because they do not allow grains to grow preferentially.^61,62^ Grain refinement is a mechanical strengthening process that raises microhardness by means of the Hall–Petch strengthening process, in which the fine grains improve the grain boundary density and prevent dislocation motion. Hence, the hardness was significantly increased from 422 HV of pure Ni coating to 1089 HV and 1055 HV of Ni-ZrO_2_ and Ni-CeO_2_ coatings, respectively.^63^ The optimal nanoparticle content of 3.5 wt% is a tradeoff between these positive effects and the prevention of agglomeration, which is found to reduce both mechanical integrity and corrosion resistance at higher loadings. This refined grain structure aids in the development of a more uniform, dense and adherent passive layer. Fine grains increase the number of grain boundaries and the grain boundary forms the fast moving diffusion channels of the formation and repair of passive oxide layers. As a result, the NiO/Ni(OH)_2_ film formed on the nanocrystalline coatings is more uniform and compact. This is clearly reflected not only in the increased impedance at low frequencies in the EIS results, but also in the post-corrosion SEM images, where the nanoparticle-reinforced surfaces appear smoother and more continuous. These findings demonstrate that a controlled dispersion with an optimized concentration of approximately 3.5 wt% CeO_2_ and ZrO2 nanoparticles enables the formation of a dense, nanocrystalline Ni-based coating with enhanced mechanical strength and corrosion resistance on the AA2219 alloy. These improvements are directly associated with the grain refinement down to ∼50 nm, dispersion strengthening, and the formation of a stable passive film in both Ni-CeO_2_ and Ni-ZrO_2_ coatings. Moreover, the Ni-CeO_2_ coating provides additional active corrosion inhibition due to the redox behavior of ceria, resulting in superior corrosion resistance. These findings offer a clear mechanism-based strategy for designing high-performance Ni-based nanocomposite coatings for aerospace and marine applications.

## 5 Conclusions

In this study, Ni-CeO_2_ and Ni-ZrO_2_ nanocomposite coatings were successfully fabricated on AA219 aluminium alloy *via* composite electrodeposition. The key outcomes of this study are as follows.

SEM and TEM analyses confirmed that an optimal concentration (3.5 wt%) and well-controlled homogeneous dispersion of CeO_2_ and ZrO_2_ nanoparticles yielded dense, uniform coatings, free from pores and cracks. This optimum introduction of nanoparticles resulted in pronounced grain refinement, reducing the Ni grain to about 50 nm, which led to a substantial enhancement in surface hardness up to 1089 HV (158%) and 1055 HV (150%) for Ni-ZrO_2_ and Ni-CeO_2_ coatings, respectively, compared with pure Ni coating. Electrochemical measurements further demonstrated a remarkable improvement in corrosion resistance, with potentiodynamic polarization results showing a 92.6% reduction in corrosion current density for Ni-CeO_2_ while Ni-ZrO_2_ exhibited a 61.9% reduction. Consistently, EIS analysis revealed a 65-fold increase in *R*_ct_ for Ni-CeO_2_, confirming the strong barrier effect provided by the uniformly dispersed nanoparticles.

### 5.1 Suggestion and future research direction

(1) In future work, the co-deposition of both ceria and zirconia results in high hardness and active inhibition.

(2) Investigating the long-term stability of coatings and different electrolyte concentrations will further validate for different harsh environments.

(3) In electrochemical measurement, the rate of Ce(OH)_3_ precipitation provides deep insights into active protection.

## Conflicts of interest

The authors declare that there are no conflicts of interest regarding the publication of this paper. All authors have contributed to this work according to the academic and research standards, and there are no competing interests, financial or otherwise, that could have influenced the outcomes of this study.

## Data Availability

The data supporting this article have been included as part of the manuscript.
